# Positionspapier zum Arbeiten in der Schwangerschaft auf der Intensivstation: DIVI-Empfehlungen zur Verbesserung der Lage von schwangeren Mitarbeiterinnen auf einer Intensivstation

**DOI:** 10.1007/s00063-024-01122-2

**Published:** 2024-03-26

**Authors:** Celina Cornelius, Teresa Deffner, Aileen Hill, Christina Rohlfes, Bernd Ellner, Silke Klarmann, Sabine Riedel, Sabrina Pelz, Sabrina Kopp, Laura Borgstedt, Diana Freund, Andreas Schöpfel, Patrick Meybohm, Felix Walcher, Thorsten Brenner, Stefanie Klenke

**Affiliations:** 1 Medizinische Fakultät Heidelberg, Klinik für Anästhesiologie, Universität Heidelberg, Im Neuenheimer Feld 420, 69120 Heidelberg, Deutschland; 2 Klinik für Anästhesiologie und Intensivmedizin, Universitätsklinikum Jena, Jena, Deutschland; 3 Klinik für Anästhesiologie und Klinik für Operative Intensivmedizin, Uniklinik RWTH Aachen, Aachen, Deutschland; 4 BDH-Klinik Hessisch Oldendorf, Hessisch Oldendorf, Deutschland; 5 Klinik rechts der Isar, Technische Universität München, München, Deutschland; 6 Therapiezentrum, Imland Klinik gGmbH Rendsburg, Rendsburg, Deutschland; 7 Physikalische Therapie und Rehabilitation, Universitätsklinikum Leipzig, Leipzig, Deutschland; 8 Universitäts- und Rehabilitationskliniken Ulm, Ulm, Deutschland; 9 Zentrum für Kardiologie, Kardiologie I, Universitätsmedizin Mainz, Mainz, Deutschland; 10 TUM School of Medicine, Klinik für Anästhesiologie und Intensivmedizin, Klinikum rechts der Isar, Technische Universität München, München, Deutschland; 11 Klinik für Anästhesie, Intensiv‑, Notfall- und Schmerzmedizin, Universitätsmedizin Greifswald, Greifswald, Deutschland; 12 Betriebsärztlicher Dienst, Universitätsklinikum Würzburg, Würzburg, Deutschland; 13 Klinik und Poliklinik für Anästhesiologie, Intensivmedizin, Notfallmedizin und Schmerztherapie, Universitätsklinikum Würzburg, Würzburg, Deutschland; 14 Universitätsklinik für Unfallchirurgie A.ö.R, Universitätsklinikum Magdeburg, Magdeburg, Deutschland; 15 DIVI e.V., Berlin, Deutschland; 16 Klinik für Anästhesiologie und Intensivmedizin, Universitätsklinikum Essen, Essen, Deutschland

**Keywords:** Mutterschutz, Beschäftigungsverbot, Personalplanung, Weiterbeschäftigung, Ressourcen, Maternity protection, Prohibition of employment, Human resource planning, Ongoing employment, Ressources

## Abstract

Das Mutterschutzgesetz soll die Mutter und das Kind vor Gefahren, Überforderung und Gesundheitsschädigung am Arbeitsplatz ebenso wie vor finanziellen Einbußen und dem Verlust des Arbeitsplatzes schützen. Allerdings werden die mit dem Mutterschutzgesetz definierten Ziele der Sicherheit und Gesundheit der schwangeren Mitarbeiterin einerseits und der Verhinderung von Benachteiligungen im Berufsleben andererseits derzeit auf der Intensivstation noch nicht ausreichend erfüllt. Es sollte eine für alle Beteiligten gewinnbringende Umsetzung des Mutterschutzgesetzes auch in den in der DIVI vertretenen Fachbereichen vorangetrieben werden, insbesondere das Arbeiten von Schwangeren im ärztlichen und Pflegefachpersonal sowie in weiteren Gesundheitsfachberufen (Atmungstherapie, Physiotherapie, Logopädie, Psychologie und Sozialdienst) auf Intensivstationen. Es soll dafür sensibilisiert werden, jede schwangere und stillende Mitarbeiterin individuell zu betrachten und gemeinsam eine persönliche Lösung für die Weiterbeschäftigung auch während der Schwangerschaft auf der Intensivstation zu erarbeiten. Es werden mögliche Wege und Lösungen zur Erreichung dieses Ziels skizziert und Praxisbeispiele zur Umsetzung im klinischen Alltag gegeben. Diese basieren auch auf einer umfangreichen Darstellung von Tätigkeiten nach einem Ampelsystem für alle Berufsgruppen. Aspekte, die gegen die Beschäftigung einer schwangeren Mitarbeiterin auf der Intensivstation sprechen oder diese einschränken, werden diskutiert und potenzielle Lösungsoptionen aufgezeigt.

**Zusatzmaterial online:**

Die Online-Version dieses Beitrags (10.1007/s00063-024-01122-2) enthält tabellarische Übersichten über die Tätigkeiten der Berufsgruppen pflegerischer Dienst, Physiotherapie, Logopädie und Atmungstherapie.

## Abstract (en)

The Maternity Protection Act is intended to protect the mother and the child from hazards, excessive demands and damage to health in the workplace, and from financial disadvantages and loss of employment. However, the objectives defined by the Maternity Protection Act—the safety and health of the pregnant employee on the one hand and the prevention of disadvantages in working life on the other—are not yet adequately achieved in the intensive care unit (ICU). Implementation of the Maternity Protection Act to the benefit of all involved parties should also be promoted in the specialist areas represented by the DIVI, in particular the work of pregnant physicians and nursing staff and other working specialists (respiratory therapists, physiotherapists, speech therapists, psychotherapists, and social workers) in the ICU. The aim of this paper is to raise awareness of the need to consider each pregnant and breastfeeding staff member individually and to work together to find a personal solution for continuing to work in the ICU. Possible ways and solutions to achieve this goal are outlined and practical examples are given for implementation in everyday clinical routine. These are also based on comprehensive presentation of activities according to a traffic light color-code system for all occupational groups. Arguments against pregnant employees working in the ICU are discussed and possible solutions are presented

## Infobox

Dieses Positionspapier wurde für die Arbeitsgruppe „Arbeiten in der Schwangerschaft auf der Intensivstation“ der Deutschen Interdisziplinären Vereinigung für Intensiv- und Notfallmedizin (DIVI e.V.) unter Leitung der Jungen DIVI und in Zusammenarbeit mit der Sektion Pflegeforschung und Pflegequalität und der Sektion Therapeutische Gesundheitsfachberufe erstellt.

## Einleitung

Das Thema „Arbeiten einer schwangeren Mitarbeiterin im Krankenhaus“ findet in den letzten Jahren erfreulicherweise immer mehr Aufmerksamkeit, auch durch die Novellierung des Mutterschutzgesetzes im Jahre 2018 [1]. Das Mutterschutzgesetz soll die Mutter und das werdende Kind vor Gefahren, Überforderung und Gesundheitsschädigung am Arbeitsplatz, ebenso wie vor finanziellen Einbußen und dem Verlust des Arbeitsplatzes schützen und sieht nach Aktualisierung ein vermehrtes „Mitspracherecht“ der schwangeren Mitarbeiterin vor [2]. Allerdings werden die mit dem Mutterschutzgesetz definierten Ziele der Sicherheit und Gesundheit der schwangeren Mitarbeiterin einerseits und der Verhinderung von Benachteiligungen im Berufsleben andererseits derzeit noch nicht erreicht [3, 4]. Zu häufig werden pauschal betriebliche Beschäftigungsverbote ausgesprochen, ohne die individuelle Situation der schwangeren Mitarbeiterin ausreichend zu betrachten.

Eine für alle Beteiligten gewinnbringende Umsetzung des Mutterschutzgesetzes soll in den in der DIVI vertretenden Fachbereichen vorangetrieben werden. Der Berufsverband Deutscher Anästhesistinnen und Anästhesisten e. V. (BDA) [5] sowie die Deutsche Gesellschaft für Orthopädie und Unfallchirurgie (DGOU; [6, 7]) haben bereits Stellungnahmen zum Arbeiten von schwangeren Ärztinnen formuliert und in einem kürzlich publizierten Artikel wurden Hinweise zum Arbeiten von schwangeren Ärztinnen in der Anästhesie dargelegt [8]. Somit gibt es erste positive Signale für eine Umsetzung des Mutterschutzgesetzes bei Ärztinnen in der Anästhesie. Nachfolgend soll die Aufmerksamkeit jedoch auf die Intensivmedizin gelegt werden, insbesondere auf die folgenden 2 großen Bereiche:

1. Arbeiten von schwangeren Ärztinnen auf einer Intensivstation: Eine Schwangerschaft führt in vielen Fällen bei ärztlichen Mitarbeiterinnen zu einem Ausschluss von Tätigkeiten auf der Intensivstation.
2. Berücksichtigung von schwangerem Personal der Gesundheitsfachberufe, insbesondere des Pflegefachpersonals sowie der Atmungstherapie, Physiotherapie und Logopädie auf einer Intensivstation. Für diese Mitarbeiterinnen wird häufig pauschal ein Beschäftigungsverbot ausgesprochen

Ein pauschales Beschäftigungsverbot für schwangere Mitarbeiterinnen aller Berufsgruppen auf einer Intensivstation ist nicht gerechtfertigt und nicht adäquat.

## Ziel und Zweck des Positionspapiers

In diesem Positionspapier sollen die KlinikdirektorInnen, ChefärztInnen, Pflege- und Stationsleitungen, Therapieleitungen, BetriebsärztInnen, Vorstände der Kliniken, Gewerbeaufsichten der einzelnen Bundesländer und weitere beteiligte Interessensgruppen dafür sensibilisiert werden, jede schwangere Mitarbeiterin sowie ihren Arbeitsplatz und Tätigkeitsbereich individuell zu betrachten und gemeinsam eine personalisierte Lösung für das Weiterarbeiten auf einer Intensivstation zu finden. Es sollen mögliche Wege und Lösungen zur Erreichung dieses Ziels skizziert werden. Das Arbeiten von schwangeren Mitarbeiterinnen auf der Intensivstation sollte nicht als unmöglich betrachtet werden, sondern realisierbar erscheinen und in die Praxis umgesetzt werden können. Bei Meldung einer Schwangerschaft seitens einer Mitarbeiterin auf der Intensivstation sollte nicht die Frage gestellt werden, ob das Weiterarbeiten überhaupt möglich ist, sondern wie es konkret gestaltet werden kann. In diesem Positionspapier liegt der Schwerpunkt auf der praktischen Umsetzung des Mutterschutzgesetzes für die schwangere Mitarbeiterin. In dem Kapitel „Rückkehr nach Mutterschutz/Elternzeit“ werden die Besonderheiten der stillenden Mitarbeiterin auf der Intensivstation adressiert.

## Praktische Umsetzungen des Mutterschutzgesetzes

**Figure 1:**
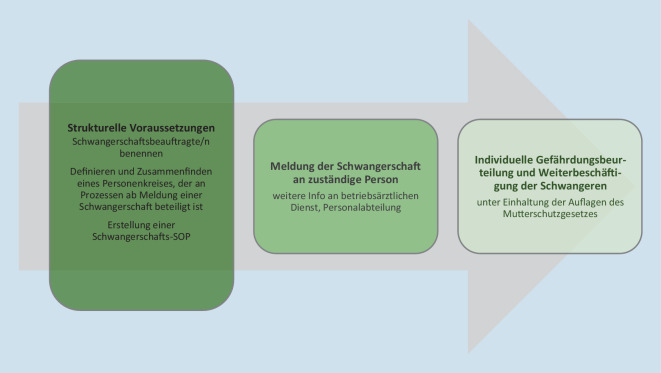

Abb. 1 gibt einen Überblick über die möglichen Schritte, die das Arbeiten von schwangeren Mitarbeiterinnen auf der Intensivstation ermöglichen können.

### 1. Strukturen/Grundvoraussetzungen schaffen

#### Benennung eines/r Schwangerschaftsbeauftragten.

Jedes Krankenhaus sollte eine/n Beauftragte/n für den Mutterschutz bestellen. Es ist empfehlenswert, dass diese/r über eine ausreichende und aktuelle Sachkunde (z. B. über Publikationen und Gesetzestexte) verfügt. Zudem ist es ratsam, dass es jeweils eine/n Beauftragte/n für die entsprechenden Berufsgruppen gibt, da nur diese Person den berufsspezifischen Arbeitsplatz kennt und aufgrund dieser Expertise eine individuelle Gefährdungsbeurteilung (s. unten) vornehmen kann. Die/der Beauftragte soll die Meldung der Schwangeren aufnehmen, die Schwangere über die jeweiligen Bestimmungen aufklären und sie auf ihrem weiteren Weg begleiten.

#### Definieren und Zusammenstellen eines Personenkreises, der an den Prozessen ab Meldung einer Schwangerschaft beteiligt ist.

Eine weitere wichtige Vorarbeit, die jedes Krankenhaus zu leisten hat, ist das Definieren und Zusammenstellen eines Personenkreises, der an den Prozessen rund um das Thema „Arbeiten in der Schwangerschaft“ beteiligt ist. Dieser Personenkreis kann je nach Krankenhaus aus betriebsärztlichem Dienst, sicherheitstechnischem Dienst, ggf. Personalrat, Schwangerschaftsbeauftragten und Krankenhausleitung oder weiteren Personen bestehen. Es ist unerlässlich, dass sich dieser Personenkreis regelmäßig zusammenfindet, austauscht und einen gemeinsamen Konsens erzielt, wie das Arbeiten von schwangeren Mitarbeiterinnen in ihrem Krankenhaus (und auch auf der Intensivstation) umgesetzt werden kann.

#### Gemeinsames Erstellen einer SOP, die die Abläufe ab Meldung einer Schwangerschaft regelt.

Nachfolgendes Ziel sollte die gemeinsame Erstellung einer Standard Operating Procedure (SOP) sein, die im Konsens mit dem oben genannten Personenkreis erstellt wird. Diese SOP soll die Abläufe ab Meldung einer Schwangerschaft im Krankenhaus/in der Abteilung zusammenfassen. Dazu gehören auch eine Gefährdungsbeurteilung und die Definition von Tätigkeiten, die möglich bzw. ausgeschlossen sind.

### 2. Meldung der Schwangeren an beauftragte Person

Sobald die Meldung einer Schwangerschaft eingeht, müssen Arbeitgebende unverzüglich Schutzmaßnahmen im Rahmen des Mutterschutzgesetzes ergreifen und mit der Schwangeren schriftlich festhalten. Des Weiteren ist eine umfängliche Aufklärung über die Anpassungen der Arbeitsbedingungen nach § 10 Abs. 2 MuSchG mit der Schwangeren in einem persönlichen Gespräch erforderlich. Es erfolgt weiterhin die Meldung der Schwangerschaft an den betriebsärztlichen Dienst, sowie an die Personalabteilung. Die Personalabteilung meldet die Schwangerschaft unter anderem auch beim Landesamt für Finanzen.

### 3. (Individuelle) Gefährdungsbeurteilung

Generell ist es notwendig, dass jedes Krankenhaus anlassunabhängig nach § 5 des Arbeitsschutzgesetzes die unterschiedlichen Arbeitsplätze auf potenziell gesundheitsgefährdende Einflussfaktoren prüft. Dies wird als allgemeine Gefährdungsbeurteilung für alle Tätigkeiten und potenzielle Arbeitsplätze bezeichnet.

Nach Meldung der Schwangerschaft wird gemeinsam mit der dafür beauftragten Person eine individuelle Gefährdungsbeurteilung durchgeführt. Die Initiative zur Durchführung dieser individuellen Gefährdungsbeurteilung muss vom Arbeitgeber (delegiert an die beauftragte Person) ausgehen und darf keinesfalls der schwangeren Mitarbeiterin auferlegt werden.

Die schwangere bzw. stillende Mitarbeiterin kann erwarten, dass die gesetzlichen Vorschriften beachten werden. Deswegen werden gemäß Mutterschutzgesetz die Arbeitsbedingungen so gestaltet, dass die schwangere oder stillende Frau und ihr Kind ausreichend vor physischen oder psychischen Beeinträchtigungen geschützt werden. Dabei sollte besonders auch der jeweilige Ausbildungsstatus und die Wünsche der schwangeren Mitarbeiterin berücksichtigt werden. Zudem müssen die Arbeitsbedingungen und damit einhergehend die Gefährdungsbeurteilung fortlaufend vom Arbeitgeber überprüft und ggf. angepasst werden. Diese Beurteilung wird an den betriebsärztlichen Dienst weitergeleitet, der seinerseits eine Prüfung durchführt. Bestenfalls gelingt diese unter Berücksichtigung der im Vorfeld gemeinsam erstellen SOP zu diesem Thema. Neben der Prüfung obliegt dem betriebsärztlichen Dienst die Beratung der Schwangeren in Bezug auf Impfstatus und weitere arbeitsmedizinische Themen. Die Aufsichtsbehörden urteilen über eine „unzumutbare Gefährdung“ und somit über die Notwendigkeit eines betrieblichen Beschäftigungsverbots. Ihr Urteil über die Weiterbeschäftigung der schwangeren Ärztin ist bindend [5]. Anhand der Ergebnisse einer Umfrage von Marburger Bund, Deutscher Ärztinnenbund, der Initiative Operieren in der Schwangerschaft, der Deutschen Gesellschafft für Orthopädie und Unfallchirurgie und des Verbands der Chirurginnen aus dem Jahre 2023 [4] wird erkenntlich, dass es hier noch deutlichen Optimierungsbedarf gibt: Bei nur durchschnittlich 61 % der Teilnehmerinnen lag eine allgemeine Gefährdungsbeurteilung vor und nur bei zwei Drittel lag eine individuelle Gefährdungsbeurteilung vor [4]. Unabhängig von den betrieblichen Arbeitsbedingungen kann der individuelle Gesundheitszustand in der Schwangerschaft oder nach der Entbindung ein ärztliches Beschäftigungsverbot rechtfertigen (§ 16 MuSchG). Dabei obliegt dem/den behandelnden ÄrztInnen ein Entscheidungsspielraum, ob ein teilweises (zeitlich befristetes/aufgabenbezogenes/vorläufiges) oder ein vollumfängliches Beschäftigungsverbot ausgesprochen wird. Ebenfalls kann ein Beschäftigungsverbot auch seitens des Arbeitsgebers ausgesprochen werden, sofern keine Arbeitsmöglichkeit geschaffen werden kann, die für den Schutz der schwangeren Mitarbeiterin und des ungeborenen Lebens erforderlich ist.

## Arbeitszeitlicher Gesundheitsschutz und Schutzfristen

Da das Verbot des Arbeitens zu bestimmten Zeiten und in bestimmten Situationen für alle Berufsgruppen gleichermaßen gültig ist, stellen wir dies für alle Berufsgruppen gemeinsam dar.

### Schutzfristen vor und nach der Entbindung

- Werdende Mütter dürfen in den letzten 6 Wochen vor der Entbindung nicht beschäftigt werden. Eine ausdrückliche Erklärung zur Arbeitsleistung ist möglich und kann jederzeit widerrufen werden (§ 3 Abs. 1 MuSchG)
- Nach der Entbindung dürfen Mütter bis zum Ablauf von 8 Wochen nicht beschäftigt werden (§ 3 Abs. 2 MuSchG), 12 Wochen bei Frühgeburten, Mehrlingsgeburten und wenn vor Ablauf von 8 Wochen nach der Entbindung bei dem Kind eine Behinderung (§ 2 Abs. 1 Satz 1 SGB IX) ärztlich festgestellt wird

### Mehrarbeit, Nachtarbeit und Ruhezeit, Sonn- und Feiertagsarbeit

#### Mehrarbeit und Nachtarbeit

- Reguläre werktägliche Dienstzeiten: 6:00–20:00 Uhr
- Zulässig sind Arbeitszeiten von max. 8,5 h pro Tag oder 90 h in der Doppelwoche
- Die Nachtarbeit einer schwangeren oder stillenden Frau zwischen 20:00 Uhr und 6:00 Uhr ist grundsätzlich verboten (§ 5 MuSchG). In besonders begründeten Einzelfällen kann die Aufsichtsbehörde gemäß § 29 Abs. 3 Ziff. 1 MuSchG Ausnahmen bewilligen (Details s. *Rechtsprechung, cave: Genehmigung der Aufsichtsbehörde ausdrücklich erforderlich*)
- Unter engen Voraussetzungen Arbeit zwischen 20:00 Uhr und 22:00 Uhr möglich (§ 5 Abs. 1 Satz 2), (Details s. Rechtsprechung, cave: Genehmigung der Aufsichtsbehörde erforderlich; solange die Aufsichtsbehörde den Antrag nicht ablehnt oder die Beschäftigung zwischen 20:00 Uhr und 22:00 Uhr nicht vorläufig untersagt, darf die Frau beschäftigt werden. Lehnt die Aufsichtsbehörde den Antrag nicht innerhalb von 6 Wochen nach Eingang des vollständigen Antrags ab, gilt die Genehmigung als erteilt.)

#### Ruhezeit

Nach Beendigung der täglichen Arbeitszeit muss der schwangeren/stillenden Frau eine ununterbrochene Ruhezeit von mindestens 11 h gewährt werden (§ 4 Abs. 2 MuSchG).

#### Sonn- und Feiertagsarbeit

- Die Arbeit an Sonn- und Feiertagen ist für Schwangere und Stillende grundsätzlich verboten (§ 6 MuSchG)
- Eine Beschäftigung ist aber möglich, wenn sich die Frau dazu ausdrücklich bereit erklärt (Anmerkung: Die Erklärung kann jederzeit widerrufen werden) und verschiedene weitere Voraussetzungen erfüllt sind (Details s. Rechtsprechung)

Die Grundvoraussetzung bei jeglichen Arbeitszeiten/-modellen ist, dass die schwangere Mitarbeiterin nicht Alleindiensthabende ist.

## Ausübung von Tätigkeiten nach Ampelsystem

Grundlage für die Überlegungen zum Einsatz einer schwangeren Mitarbeiterin auf der Intensivstation können Positivlisten sein, die als unbedenklich erachtete Tätigkeiten und Einsatzgebiete aufführen. Es sollten ebenfalls transparente Informationen und genaue Kenntnisse über primär unzulässige Tätigkeiten und Arbeitsbedingungen einer schwangeren Mitarbeiterin auf der Intensivstation vorliegen. Letztere sind nicht mit dem Mutterschutzgesetz vereinbar und sollen/dürfen/können von einer schwangeren Mitarbeiterin auf der Intensivstation nicht durchgeführt werden, da sie eine Beeinträchtigung für das Leben des ungeborenen Kinds oder die Mutter bedeuten könnten. Zusätzlich gibt es Tätigkeiten, die in Abhängigkeit von dem Erfahrungsschatz der schwangeren Mitarbeiterin und von lokalen Besonderheiten zu betrachten sind. Hier sollte eine individuelle Absprache mit der schwangeren Mitarbeiterin erfolgen. Ärztinnen können – nach Ansicht zahlreicher betriebsärztlicher Dienste – während der Schwangerschaft Tätigkeiten wie z. B. das Legen und Wechseln eines zentralen Venenkatheters und Bronchoskopien durchführen, wenn sie darin eine ausreichende Expertise haben und dies als für sie zumutbar beurteilen. Die darin ungeübte Ärztin sollte diese Tätigkeiten nicht während ihrer Schwangerschaft auf der Intensivstation erlernen. Die Angabe von invasiven Tätigkeiten bezieht sich ausdrücklich auf PatientInnen mit negativem Infektstatus bzw. ohne Anhalt für eine vorliegende übertragbare Infektion, bei der von keiner Gefährdung für Mutter und ungeborenes Leben auszugehen ist.

Mit Wegfall der SARS-CoV-2-Arbeitsschutzverordnung im Februar 2023 sind die besonderen gesetzlichen Vorgaben zum Infektionsschutz gegen SARS-CoV‑2 entfallen. ArbeitgeberInnen müssen nun bezüglich dieser Infektionskrankheit im Rahmen des gesetzlichen Arbeitsschutzes – am besten mit dem Personenkreis, der an den Prozessen ab Meldung einer Schwangerschaft beteiligt ist – prüfen, ob und welche Maßnahmen zum Infektionsschutz am Arbeitsplatz erforderlich sind. Zur Unterstützung hat das Bundesministerium für Arbeit und Soziales Empfehlungen zum betrieblichen Infektionsschutz vor COVID-19, Influenza und auch Erkältungskrankheiten veröffentlicht [9].

**Figure 2:**
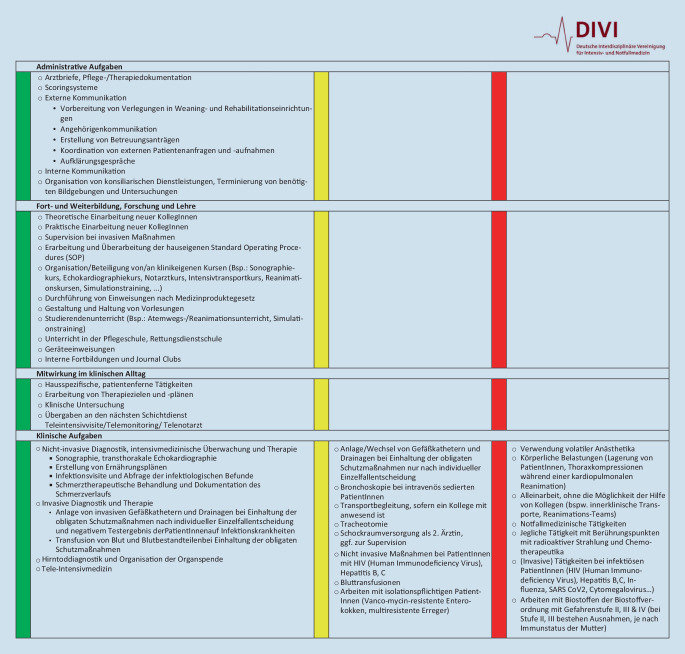

Abb. 2 gibt einen Überblick über die Tätigkeiten nach einem Ampelprinzip für die Berufsgruppe ärztlicher Dienst. Im Onlinezusatzmaterial finden sich tabellarische Übersichten für die Berufsgruppen pflegerischer Dienst, Physiotherapie, Logopädie und Atmungstherapie. Für die Mitarbeiterinnen der Psychologie und des Sozialdiensts wird auf die Darstellung einer tabellarischen Übersicht für die Tätigkeiten verzichtet, da diese weitestgehend ihren bisherigen Tätigkeiten nachgehen können. Die Tabellen dienen als Orientierung bzw. Entscheidungshilfe für die schwangere Mitarbeiterin und sämtliche im Prozess eingebundenen Personen. Die Tabellen sind gegliedert in Tätigkeiten in der Klinik (administrative Aufgaben, Mitwirkung im klinischen Alltag, klinische Aufgaben) und Tätigkeiten in Fort- und Weiterbildung sowie Lehre und Forschung. Als wissenschaftliche Dachgesellschaft unterstützt die DIVI jegliche Forschungs- und Lehraktivitäten, da sie auch der Schwangeren ein breites Feld einer gefahrlosen Weiterbeschäftigung ermöglichen und die Chance bieten den Wissensstand zu erhalten und auszubauen. Anzumerken ist, dass die Listen keinen Anspruch auf Vollständigkeit erheben und daher nicht abschließend sind. Aus der exemplarischen Darstellung ergeben sich keine Ansprüche oder Forderungen. Die Maßnahmenempfehlungen müssen immer nach individuellen und lokalen Gegebenheiten und Vorgaben im Gesamtkontext betrachtet werden.

## Umsetzung im klinischen Alltag

Nach den bisherigen allgemeinen Hinweisen zu der praktischen Umsetzung erscheint es wichtig, konkrete Abläufe oder Praxisbeispiele darzustellen.

### Praxisbeispiel „Strukturen schaffen“

In einem Krankenhaus mit einer 20-Betten-Intensivstation erhalten alle schwangeren Mitarbeiterinnen bei Meldung der Schwangerschaft ein betriebliches Beschäftigungsverbot. Um eine Benachteiligung schwangerer und stillender Mitarbeiterinnen auf der Intensivstation zu vermeiden, wird eine anästhesiologische Oberärztin zur Schwangerschaftsbeauftragten benannt. Gemeinsam mit dem betriebs- und sicherheitstechnischen Dienst wird besprochen, wie das Arbeiten von schwangeren Mitarbeiterinnen auf dieser Intensivstation möglich ist. Der betriebsärztliche Dienst steht diesem Vorhaben initial skeptisch gegenüber. Die Schwangerschaftsbeauftrage stellt die vorhandene Literatur zu diesem Thema vor und es wird eine gemeinsame, für alle Beteiligte zufriedenstellende, Lösung gefunden. Anhand der Ergebnisse wird dann eine gemeinsame SOP erstellt, an der sich alle Beteiligten nun orientieren können.

### Praxisbeispiel „Individuelle Gefährdungsbeurteilung“

Nach Meldung der Schwangerschaft macht der/die Schwangerschaftsbeauftragte eines Krankenhauses die individuelle Gefährdungsbeurteilung für eine Intensivpflegekraft. Bei der Prüfliste der physikalischen Gefährdungen wird u. a. festgestellt, dass beim Lagern von PatientInnen die Grenzlasten für Heben und Bewegen überschritten werden. Bei den Kategorien „biologischen Gefährdungen“ und der möglichen Übertragung von Krankheiten wird festgestellt, dass häufig bei PatientInnen der infektiöse Status unbekannt ist. Zudem gibt es zahlreiche PatientInnen, die potenziell infektiös sind (z. B. positiv für Coronavirus, Influenza, multiresistente Erreger). Bei der Prüfliste zu den Arbeitsbedingungen und Arbeitsverfahren wird u. a. festgestellt, dass Nachtarbeit sowie Arbeit an Wochenenden und Feiertagen geleistet wird. Gemeinsam mit der Pflegeleitung der Intensivstation wird abgestimmt, dass eine Tätigkeit nur noch im Frühdienst stattfinden kann. Zusätzlich werden mit der schwangeren Mitarbeiterin folgende Arbeitsmöglichkeiten besprochen: Sie unterstützt bei der Pflege-/und Therapiedokumentation und der Erfassung von Variablen für Scoringsysteme. Zusätzlich übernimmt sie Aufgaben bei der theoretischen und praktischen Einarbeitung neuer KollegInnen. In der Klinik soll zudem eine neue SOP zum Delirmanagement erstellt werden, an der die schwangere Mitarbeiterin sich maßgeblich beteiligt. Zudem wird sie von der Pflegeleitung der Intensivstation in die Erstellung von Dienstplänen eingearbeitet und kann so die Pflegeleitung maßgeblich unterstützen.

### Praxisbeispiel „Ausübung von Tätigkeiten nach Ampelsystem“

Eine schwangere Fachärztin arbeitet auf der Intensivstation zur Erlangung der Zusatzbezeichnung „Spezielle Intensivmedizin“, als sie ihre Schwangerschaft meldet. Die individuelle Gefährdungsbeurteilung wird durchgeführt. Es wird besprochen, dass Sie im Früh- oder Zwischendienst (z. B. 7:00 bis 16:00 Uhr oder 11:00 bis 20:00 Uhr) arbeitet und zu keiner Zeit Alleindiensthabende auf der Station ist. Anhand der Positivliste werden gut mögliche Tätigkeiten definiert und mithilfe der Negativliste Tätigkeiten ausgeschlossen. Da die schwangere Kollegin eine erfahrene Fachärztin ist, wird mit ihr besprochen, dass Sie bei PatientInnen mit bekannt negativem Infektionsstatus arterielle und zentralvenöse Katheterwechsel sowie Bronchoskopien durchführen kann. Die ärztlichen Visiten und administrativen Aufgaben nimmt sie wie bisher wahr. Transportfahrten sowie Tätigkeiten bei infektiösen PatientInnen führen andere KollegInnen durch.

### Praxisbeispiel „Arbeitszeitlicher Gesundheitsschutz“

Die schwangere Ärztin zur Erlangung der Facharztanerkennung kann im Frühdienst arbeiten, wenn eine weitere Person (z. B.: Oberarzt/ärztin bzw. Facharzt/ärztin bei großen Intensivstationen mit doppelt besetzten Schichten) im Frühdienst vorhanden ist. Möglich wäre auch die Wahrnehmung eines Zwischendiensts (z. B. 11:00 bis 20:00 Uhr), bei dem immer gewährleistet ist, dass Früh- und Spätdienste präsent sind. Sollte nach 20:00 Uhr gearbeitet werden, muss dies bei der Aufsichtsbehörde beantragt werden. Sollte in Einzelfällen auf dieses Modell zurückgegriffen werden, muss gewährleistet werden, dass jederzeit eine weitere ärztliche Person auf der Station präsent ist (z. B. bei Intensivstationen mit gedoppelten Schichten). Die Übernahme von Nachtschichten ist nicht möglich. Das Arbeiten an Sonn- und Feiertagen ist – wie oben dargestellt – grundsätzlich verboten, kann unter verschiedenen Auflagen erwirkt werden (s. oben, Grundvoraussetzung: Die schwangere Mitarbeiterin erklärt sich dazu ausdrücklich bereit). Dies stellt sicherlich nicht das präferierte Modell dar, kann aber in Einzelfällen unter guter Absprache aller Beteiligten, Einhaltung der Gesetzgebung etc. umgesetzt werden.

### Praxisbeispiel „Schwangere Ärztin in Leitungs‑/Führungspositionen auf der Intensivstation“

Selbstverständlich können schwangere Mitarbeiterinnen in Leitungs‑/Führungsposition (z. B. Leitung einer Intensivstation) diese Funktion weiter wahrnehmen. Die Durchführung von Visiten bzw. von vielen organisatorischen Aufgaben ist problemlos möglich. Voraussetzung ist, dass für Notfallsituationen bei invasiver Tätigkeit (z. B. Reintubation, Thoraxdrainagenanlage) weiteres erfahrenes Personal vor Ort ist, zumal diese Interventionen auch bei infektiösen PatientInnen erforderlich sein können.

## Planung der Rückkehr nach Mutterschutz/Elternzeit

Bereits während der Schwangerschaft sollten der schwangeren Mitarbeiterin Optionen für ihre Rückkehr nach Mutterschutz/Elternzeit aufgezeigt werden (z. B. flexible Arbeitsmodelle, Weiterbildung in Teilzeit).

Die stillende Mitarbeiterin auf der Intensivstation: Der Arbeitgeber hat eine stillende Frau auf ihr Verlangen für die zum Stillen erforderliche Zeit, mindestens aber 2‑mal täglich für eine halbe Stunde oder einmal täglich für 1 h, freizustellen. Der Anspruch auf bezahlte Freistellung ist zeitlich bis zum 12. Lebensmonat des Kinds begrenzt (§ 7 Abs. 2 MuSchG). Die Freistellungszeiten sind weder vor- noch nachzuarbeiten und werden nicht auf die gesetzliche/tariflich vorgeschriebene Ruhepausen angerechnet.

Bei Rückkehr nach dem Mutterschutz/der Elternzeit einer noch stillenden Frau auf der Intensivstation ist folgendes zu berücksichtigen: Auch stillende Mitarbeiterinnen auf der Intensivstation benötigen nach Rückkehr aus der Elternzeit einen Arbeitsplatz nach Mutterschutzgesetz. Die praktische Umsetzung (individuelle Gefährdungsbeurteilung, Meldung an die Aufsichtsbehörde etc.) entspricht dem Vorgehen bei der schwangeren Mitarbeiterin. Die Gewährung von Stillpausen sollte auf einer Intensivstation möglich sein. Es ist zu erwarten, dass dort Räume zu Verfügung stehen, in die sich die Mitarbeiterin zurückziehen kann. Sollte die Milch mittels einer Pumpe abgepumpt werden, müssen gekühlte Lagerungsmöglichkeiten zu Verfügung stehen. Ein Kind in dieser Zeit selbst zu stillen, ist im Alltag sicherlich schwieriger zu realisieren und nur praktikabel, wenn das Kind in der Betriebskindertagesstätte untergebracht ist oder das Kind von einer Betreuungsperson in die Klinik gebracht wird.

Krankenhäuser sollten Konzepte entwickeln, wie MitarbeiterInnen, die nach ihrer Rückkehr aus Mutterschutz/Elternzeit in Teilzeit zurückkehren, auf einer Intensivstation arbeiten können. Durch den immer größeren Anteil an Frauen im ärztlichen und den anderen medizinischen Berufen (und somit auch an Frauen, die nach einer Schwangerschaft zurückkehren und ihren „normalen“ beruflichen Werdegang fortsetzten möchten), wird dies ein Thema sein, dem sich Krankenhäuser annehmen sollten. Dabei sollten selbstverständlich auch die männlichen Kollegen berücksichtigt werden, die Elternzeit wahrnehmen oder die z. B. aufgrund der Vereinbarkeit von Familie und Beruf in Teilzeit arbeiten möchten. Eine kürzliche Erhebung des Marburger Bunds zeigte, dass ein Viertel der Ärztinnen und Ärzte in NRW-Krankenhäusern in Teilzeit arbeitet [10] Ein weiteres Ziel sollte daher sein, ein familienfreundliches Arbeitsumfeld auf der Intensivstation zu etablieren.

Dies kann auf einer Intensivstation mit einem Schichtmodell schwieriger zu realisieren sein als in anderen Arbeitsumgebungen. Im Folgenden werden mögliche Schichtmodelle skizziert, wobei hervorzuheben ist, dass es eine Vielzahl individueller Schichtsysteme gibt. Für die gängigen 3‑Schicht- bzw. 4‑Schichtsysteme wäre eine Möglichkeit, eine volle Stelle auf 2 Teilzeitkräfte zu verteilen, wobei sich die Arbeitnehmerinnen zeitlich abwechseln. Weitere Lösungen sind reduzierte Anzahl der Wochenarbeitstage bei jeweils Vollarbeit pro Tag oder das Einführen von verkürzten Schichten (z. B. verkürzter Frühdienst, Zwischendienste). Es gibt sicherlich noch weitere Modelle, um das Arbeiten in Teilzeit auf einer Intensivstation erfolgreich umzusetzen, die von den lokalen Begebenheiten geprägt werden und entsprechend anzupassen sind.

Das Thema Rückkehr nach Elternzeit bzw. Arbeiten auf der Intensivstation in Teilzeit ist von großer Wichtigkeit im Kontext der Familienfreundlichkeit. Es ist allerdings zu umfassend, sodass hier nur einige Anregungen gegeben werden können.

## Mögliche Aspekte, die einer Tätigkeit schwangerer Mitarbeiterinnen auf der Intensivstation entgegenstehen

Von verschiedenen Entscheidungsträgern könnten Argumente gegen die Weiterbeschäftigung der schwangeren Mitarbeiterin (auf der Intensivstation) vorgebracht werden.

Ein mögliches Argument, das von der Leitungsebene vorgebracht werden könnte, um ein Beschäftigungsverbot zu rechtfertigen, kann die Möglichkeit der Neubesetzung der Stelle der Schwangeren sein. Diese Sichtweise erscheint allerdings als kurzsichtig. Heutzutage gibt es einen Mangel an qualifiziertem Personal der Pflege, Gesundheitsfachberufe und im ärztlichen Dienst auf der Intensivstation, sodass eine unmittelbare Neueinstellung mit gleichwertig ausgebildetem und eingearbeitetem Personal eher die Ausnahme bleiben dürfte. Zudem sollten ArbeitnehmerInnen durch eine nachhaltige Personalpolitik (also auch die schwangere Mitarbeiterin auf der Intensivstation) an das Krankenhaus als Arbeitgeber gebunden werden. Eine langfristige Personalbindung steigert die Zufriedenheit und Motivation des Teams und kann dadurch die Qualität der Patientenversorgung erhöhen.

In diesem Kontext sollte auch ein bereits vorliegender Personalmangel betrachtet werden. Hier könnten Argumente von Seiten der Entscheidungsträger (aber möglicherweise auch von KollegInnen) angeführt werden, dass nach Meldung einer Schwangerschaft nicht die gleichen Aufgaben an die Schwangere übertragen werden können wie an andere KollegInnen (wie z. B. die Durchführung von Nachtdiensten) und so ein etwaiger Personalmangel noch verschärft würde. Ebenso könnte das Argument angeführt werden, dass eine Verschiebung der Schwere der Arbeitsbelastung (die Arbeit der schwangeren Mitarbeiterin umfasst ein anderes Arbeitsspektrum als vor Bekanntgabe der Schwangerschaft) dem Grundsatz, die Arbeitsbelastung gleichmäßig unter den KollegInnen zu verteilen, widerspricht. Auch hier sollte die Leitungsebene vorausschauend denken: Eine bereits eingearbeitete, gut ausgebildete schwangere Mitarbeiterin kann den Stationsablauf maßgeblich unterstützten/entlasten und ihre Erfahrung an verschiedenen Stellen einbringen, auch wenn sich ihr Tätigkeitsspektrum ändert und nicht dem vor Meldung der Schwangerschaft entspricht. Hierbei sind die transparente und ehrliche Kommunikation im Team, Kollegialität und gemeinsame Absprachen essenziell. Dies soll auch als Signal an andere MitarbeiterInnen dienen, dass die Meldung einer Schwangerschaft nicht mit einem Beschäftigungsverbot gleichzusetzen ist.

Ebenfalls berücksichtigt werden sollte, dass die Durchführung von Tätigkeiten anhand hier dargestellter Positivlisten auf einer Intensivstation für die schwangere Mitarbeiterin nicht für alle Berufsgruppen gleichermaßen möglich ist. So ist es denkbar, dass für die schwangere Ärztin mehr Tätigkeiten anhand der Positivliste infrage kommen als z. B. für die schwangere Physiotherapeutin. Gleichfalls unterscheidet sich der zur Verfügung stehende Personalschlüssel, durch den die jeweiligen Aufgaben übernommen werden. Hierbei sollte auch die generelle Abteilungsgröße berücksichtigt werden. So kann sicherlich auf einer großen Intensivstation mit möglicherweise doppelt besetzten ärztlichen Schichten das Arbeiten einer schwangeren Ärztin besser implementiert werden als auf einer kleineren Intensivstation. Eine mögliche Lösung zur Erhaltung der Schwangeren als Arbeitskraft unter gleichzeitiger Sicherstellung der Behandlungsqualität wäre die Bildung eines „Therapeutenduos“ (z. B. in der Physiotherapie), dass die Behandlung der PatientInnen in enger Absprache gemeinsam übernimmt. Dabei sollten stets mögliche finanzielle Aspekte der Arbeitgeber (erhöhte Kosten z. B. durch „Dopplung“) den dargestellten positiven Aspekten gegenübergestellt werden.

Aus therapeutischer Sicht könnte auch argumentiert werden, dass es in Anbetracht der medizinisch-therapeutischen Komplexität der PatientInnen nicht immer sinnvoll ist, einen logischen aufeinander aufbauenden Arbeitsablauf (z. B. Befund/Assessment/Therapie/Dokumentation) auf verschiedene Personen aufzuteilen, was bei Einsatz der schwangeren Mitarbeiterin in manchen Fällen erforderlich sein kann. Dennoch können transparente Kommunikation und Absprachen dazu führen, die schwangere Mitarbeiterin für die Abteilung einzusetzen.

Auf einer Intensivstation können aufgrund der hohen Krankheitsschwere der PatientInnen Situationen entstehen, die von MitarbeiterInnen als sehr belastend empfunden werden. Solche Erfahrungen sollte eine schwangere Mitarbeiterin nicht machen müssen und es sollten dann unmittelbare Konsequenzen (z. B. Übernahme von Tätigkeiten in Lehre und Forschung) gezogen werden. Das Ziel sollte sein, nach einer Schwangerschaft rückwirkend feststellen zu können, dass in der Schwangerschaft verantwortungsvoll gehandelt wurde.

Weiterhin ist es möglich, dass andere Interessengruppen, wie z. B. der betriebsärztliche Dienst, dem Weiterarbeiten einer schwangeren Mitarbeiterin auf der Intensivstation eher restriktiv gegenüberstehen. Hier sollte der gemeinsame Dialog gesucht werden und auf publizierte Literatur zu diesem Thema und die Praktikabilität hingewiesen werden.

Auch scheint die unterschiedliche Gesetzgebung durch die Aufsichtsbehörden bzw. deren unterschiedliche Interpretation problematisch. So kann die Situation entstehen, dass Tätigkeiten in einigen Bundesländern möglich sind, während sie in den anderen Bundesländern verboten sind. Eine zukünftige Harmonisierung und einheitliche Lösung im gesamten Bundesgebiet sind wünschenswert.

Sicherlich gibt es eine Vielzahl von Argumenten, die durch am Entscheidungsprozess beteiligte Personen vorgebracht werden könnten. Hierbei ist die Relevanz des gemeinsamen Dialogs hervorzuheben, um eine für alle Beteiligten zufriedenstellende Lösung zu finden. Dabei ist mit der schwangeren Mitarbeiterin zu erörtern, was ihre Vorstellungen in Bezug auf ihre Tätigkeit in der Schwangerschaft sind. Entscheidend hierfür ist eine objektive Beurteilung mit rationaler Abwägung der Vor- und Nachteile durch kompetente Personen, die das Arbeitsumfeld der schwangeren Mitarbeiterin beurteilen können. Hier sollte auch berücksichtigt werden, dass eine Mitarbeiterin bei Meldung einer Schwangerschaft die Tätigkeit auf ihrer bisherigen Intensivstation präferenziell fortsetzen sollte, da sie dort eingearbeitet ist, die Arbeitsläufe und die KollegInnen kennt, sodass gemeinsame Absprachen besser getroffen werden können, Vertrauensverhältnisse und persönliche Beziehungen bestehen, die ein Teamgefühl fördern und somit zur Akzeptanzsteigerung der Maßnahmen positiv beitragen. Wichtig ist: Jede schwangere Mitarbeiterin und jede Schwangerschaft ist anders. So sollte individuell erörtert werden, was eine schwangere Mitarbeiterin zu leisten willens und im Stande ist. Dies kann sich im Lauf der Schwangerschaft ändern und muss dementsprechend fortwährend ergebnisoffen reevaluiert werden.

## Resümee

In diesem Positionspapier wird das Arbeiten von schwangeren Mitarbeiterinnen aus dem ärztlichen Dienst und den Gesundheitsfachberufen auf einer Intensivstation thematisiert. Es ist wünschenswert, dass sich alle Krankenhäuser mit dieser Thematik auseinandersetzen und konkrete Konzepte für ihren Standort ausarbeiten, wie – angepasst an die lokalen Gegebenheiten – das Arbeiten einer schwangeren Mitarbeiterin auf der Intensivstation ausgestaltet werden kann. Basierend auf den konkreten Handlungsempfehlungen im klinischen Alltag in diesem Positionspapier wäre eine praktische Umsetzung in naher Zukunft wünschenswert.

### Supplementary Information




