# Supplementary material for: Position paper on working in the intensive care unit during pregnancy: DIVI recommendations for improving the situation of pregnant employees in the ICU
Source: Med Klin Intensivmed Notfmed. 2024 Mar 26;119(5):364–72. [Article in German] doi: 10.1007/s00063-024-01122-2 (PMC11130002; doi:10.1007/s00063-024-01122-2)
Supplement: Supplementary file 1 [file 63_2024_1122_MOESM1_ESM.pdf]

Pflegefachfrau mit und ohne Fachweiterbildung Intensivpflege und Anästhesie (PflBG 2020 und WBVO-Pflegeberufe der Länder)

Abgeschlossene Weiterbildungen und Berufserfahrung im Bereich Intensivpflege und Anästhesie sollte als eine Expertise in der Begleitung und Durchführung der nachfolgenden Tätigkeiten verstanden werden.
Empfehlungsgrade: grün = vertretbar, gelb = individuell & eingeschränkt vertretbar, rot = nicht vertretbar
Bei der Bewertung grüner und gelber Bereiche sollte die Expertise und Erfahrung sowie ihr eigenes Sicherheitsempfinden der schwangeren Mitarbeiterin berücksichtigt werden.

Administrative Aufgaben

- Kontrolle und ggf. Durchführung der Dokumentation von Beatmungszeiten
- Kontrolle und ggf. Durchführung bzw. Anweisung der Intensivpflegefachperson aller
- Assessments/Scorings (z.B. Nummerische Rating Skala (NRS), Richmond Agitation Sedation Scale (RASS), Confusion Assessment Method für Intensivstation (CAM-ICU), Risikoskalen zu Dekubitus, Ernährung, Sturz…)
- Vorbereiten von Medikamentenverordnungen mit entsprechenden Schutzmaßnahmen, ausgenommen Zytostatika
- Schreiben von Intensivtagebucheinträgen
- Bestellvorgänge von Medikamentenverordnungen, Büroartikel und
- Verbrauchsmaterial, Bettbestellungen
- Externe Kommunikation
  - Vorbereitung der Unterlagen von Verlegungen in- und externe Institutionen
  - Angehörigenkommunikation z.B. Angehörigentelefonat 1x täglich, Koordination der Besucherregelungen
  - Beratungsgespräche im Pflegeprozess, Pflegebedürftigkeit, Professionelle Pflegeberatung
- Interne Kommunikation
  - Bearbeiten von Labor/- und Mikrobiologieanforderungen
  - Administrative Aufnahme- und Verlegungsvorgänge
- Durchführen von vorbereiteten Exceltabellen für Auswertungszwecke zum Ausfüllen

Fort- und Weiterbildung, Forschung und Lehre

- Einweisung in Geräte der Intensivstation (sofern entsprechende Qualifikation als Hersteller-/Anwendungseinweiser vorliegt)
- Einweisung in Softwareprogramme, digitale Patientenakte
- Theoretische Einarbeitung neuer KollegInnen
- Supervision bei invasiven Maßnahmen
- Präsentieren der Intensivstation z.B. herumführen und informieren
- Erarbeitung und Überarbeitung des Standards/Verfahrensanweisung (VA)/ Standard Operating Procedures (SOPs) nach Qualitätsmanagement- (QM-) Richtlinien und Evidenz
- Lehre an Universität/Hochschule /Berufsfachschulen/-akademien
- Interne Fortbildungen und Journal Clubs, Fallbesprechungen, Ethische Fallbesprechungen
- Erstellung von (One-Minute-Wonder-Fortbildung) OMWs unter Berücksichtigung der Evidenz
- Unterstützung im Projektmanagement/Changemanagement

<u>Pflegewissenschaftliche Tätigkeiten:</u><br>○ Erstellen und Evaluieren von Surveillanceparametern<br>○ Evaluieren von Qualitätsindikatoren<br>○ Qualitative und quantitative Auswertungen<br>○ Formulierung von wissenschaftlichen Fragestellungen<br>○ Publikationen<br>○ Ethikanträge<br>○ Mitarbeit in Gremien/Task Forces<br>○ Edukation		
**Mitwirkung im klinischen Alltag**		
<u>Pflegefachfrau</u><br>○ Erarbeitung des Pflegeprozesses und Pflegeplanung Level of Care 1<br>○ Anleitung und Durchführung von Atemübungen<br>○ Allgemeine Übergaben<br><u>Fachweiterbildung Intensivpflege und Anästhesie, ab Level of Care 3 mit Advanced Practice Nurse</u><br>○ Erarbeitung des Pflegeprozesses und Pflegeplanung ab Level of Care 2<br>○ Teleintensivpflegevisite/Telemonitoring<br>○ Planung und Evaluation von Ernährungsplänen	<u>Fachweiterbildung Intensivpflege und Anästhesie, ab Level of Care 3 mit Advanced Practice Nurse</u><br>○ Klinische Untersuchungen	
**Klinische Aufgaben**		
<u>Fachweiterbildung Intensivpflege und Anästhesie</u><br>○ Evaluation und Dokumentation des Schmerzverlaufs<br>○ Überwachung und Evaluation von Nierenersatzverfahren<br>○ Evaluation von Blutgasanalysen und Anpassung der Beatmung<br>○ Evaluation und Anpassung des Fluidmanagements<br>○ Evaluation und Anpassung des Sedierungsmanagements<br>○ Evaluation und Anpassung des Kreislaufmanagements<br>○ Anleitung und Durchführung von Atemübungen	<u>Fachweiterbildung Intensivpflege und Anästhesie</u><br>○ Ab Level of Care 2 nachweislich ohne Infektionskrankheiten, multiresistenten Erregern, Chemotherapie/Zytostatikatherapie, Applikation intra – und post- Narkosegasen, möglichem aggressionspotenzial	○ Körperliche Belastungen (Lagerung von PatientInnen, Thoraxkompressionen während einer Reanimation)<br>○ Alleinarbeit, ohne die Möglichkeit der Hilfe von Kollegen (bspw. Teilnahme an innerklinischen Transporten oder Reanimations-Teams)<br>○ Notfallmedizinische Tätigkeiten<br>○ Jegliche Tätigkeit mit Berührungspunkten mit<br>• Level of Care ab 2 mit Sekretmanagement jeglicher Art<br>• Level of Care ab 2 mit Beatmung jeglicher Art<br>• Level of Care ab 2 mit Weaningverfahren jeglicher Art<br>• Level of Care ab 3 mit extrakorporalen Verfahren jeglicher Art<br>• Level of Care ab 2/3 mit High Flow/non-invasiver/invasiver Beatmung<br>○ Positionierungen und Mobilisation von Level of Care ab 2 mit Fokus zur Stabilisierung der Lunge, des Kreislaufs und des Bewusstseins<br>○ Alle aerosolbildenden Interventionen<br>○ Herstellung von Medikamenten<br>○ Vorbereiten von Transfusionen<br>○ Alle Tätigkeiten, die auf der Negativliste der Arbeitssicherheit stehen

Physiotherapeutin

Empfehlungsgrade: grün = vertretbar, gelb = individuell & eingeschränkt vertretbar, rot = nicht vertretbar
Bei der Bewertung grüner und gelber Bereiche sollte die Expertise und Erfahrung sowie ihr eigenes Sicherheitsempfinden der schwangeren Mitarbeiterin berücksichtigt werden.

**Administrative Aufgaben**		
o Therapieplanung<br>• Die PatientInnenanzahl sollte bei einer Vollkraft-Stelle 10 PatientInnen nicht überschreiten, um ausreichende Pausenzeiten gewährleisten zu können. Wegezeiten sollten beachtet werden, wenn die Kolleginnen zentral verplant werden.<br>o Erarbeitung von Verfahrensanweisung (VA)/ Standard Operating Procedures (SOPs)<br>o Mitarbeit bei (Re)Zertifizierungen (Stroke Unit, Weaning-Zentrum, usw.)<br>o Erstellen und Durchführen von Schulungen von (neuen) MitarbeiterInnen (Therapie, Pflege, ärztlicher Dienst, Hilfskräfte) zu berufseigenen Themen im Haus<br>o Konzepterstellung, Literaturrecherche		
**Fort- und Weiterbildung, Forschung und Lehre**		
o Supervision und Anleitung sowie Ansprechpartnerin von Auszubildenen, StudentInnen, HospitantInnen, PraktikantInnen<br>o Anleitung/Schulung		
**Mitwirkung im klinischen Alltag**		
o Dokumentationsarbeiten: bspw. Erstellen von Berichten<br>o Angehörigenkommunikation<br>o Hausspezifische, patientenferne Tätigkeiten		
**Klinische Aufgaben**		
o Wahrnehmungsschulung<br>• z.B.: Schulung der Oberflächensensibilität, Propriozeptionsschulung (mit Einschränkungen) ohne passives oder assistives Bewegen der PatientInnen<br>o Atemtherapie<br>• Passive Maßnahmen ohne Veränderung der Ausgangsstellung der PatientInnen<br>• Anleitung und Durchführung von Therapieeinheiten mit Atemtrainer<br>• Edukation: z.B.: Atemwahrnehmung, Atemlenkung, Anleitung von Eigenübungen<br>o Manuelle Lymphdrainage<br>• Entstauende Maßnahmen die kein Umlagern der PatientInnen erfordern und kompressorische Maßnahmen mit Hilfe von KollegInnen	o Cardiopulmonales Training (Einsatz von Trainingsgeräten wie z.B. Bettfahrrad bei Einrichtung und Abbau durch Kollegen)<br>o Atemtherapie<br>• Hustentechniken<br>o Edukation: Bewegungsschulung, Eigenübungen anleiten	o Frühmobilisation<br>o Lagerung, Vertikalisierung<br>o Alleinarbeit, ohne die Möglichkeit der Hilfe von KollegInnen<br>o Tätigkeiten bei infektiösen PatientInnen

Logopädin (synonym sind hier auch Kolleginnen mit den Berufsbezeichnungen Sprachtherapeutin, Klinische Linguistin gemeint).

Abgeschlossene Weiterbildungen und Berufserfahrung im Bereich Intensivmedizin sollte als eine Expertise in der Begleitung und Durchführung der nachfolgenden Tätigkeiten verstanden werden.
Empfehlungsgrade: grün = vertretbar, gelb = eingeschränkt vertretbar, rot = nicht vertretbar.
Bei der Bewertung grüner und gelber Bereiche sollte die Expertise und Erfahrung sowie ihr eigenes Sicherheitsempfinden der schwangeren Mitarbeiterin berücksichtigt werden.

grün	gelb	rot
**Administrative Aufgaben**		
○ Auswerten und Schreiben von Befunden sowie Überleit-/Entlassbriefen<br>○ Bei bereits etablierten weiteren Tätigkeiten (außerhalb der therapeutischen Arbeit):<br>• Abteilungsleitungsfunktionen<br>• Teilnahme an Besprechungen<br>• Mitarbeit in Arbeitsgruppen/Gremien<br>• Arbeit als Medizinproduktebeauftrage<br>• Mitarbeit im klinischen Ethikkomitee<br>○ Angehörigenkommunikation<br>○ Mitarbeit bei (Re-)Zertifizierungen (Stroke Unit, Weaning-Zentrum, usw.)		
**Fort- und Weiterbildung, Forschung und Lehre**		
○ Erarbeitung von Verfahrensanweisung (VA)/ Standard Operating Procedures (SOPs)<br>○ Erstellen/ Durchführen von Schulungen von (neuen) MitarbeiterInnen (Therapie, Pflege, ärztlicher Dienst, Hilfskräfte) zu berufseigenen Themen im Haus<br>○ Supervision und Anleitung<br>○ Ansprechpartnerin, Supervision und Anleitung von HospitantInnen, PraktikantInnen, Auszubildenen, SchülerInnen und StudentInnen<br>○ Hausspezifische, patientenferne Tätigkeiten<br>○ Unterstützung bei wissenschaftlichen Tätigkeiten		
**Mitwirkung im klinischen Alltag**		
○ Hausspezifische, patientenferne Tätigkeiten<br>○ Therapieplanung		
**Klinische Aufgaben**		
○ Dysphagietherapie<br>• Klinische Diagnostik, Tätigkeiten und Methoden, ohne erhöhte Exposition<br>• Therapeutische Nahrungsgaben und Esstraining<br>○ FEES (flexible endoskopische Evaluation des Schluckens):<br>• mit FEES-Zertifikat und unter Einhaltung von weiteren Schutzmaßnahmen bei bspw. nicht infektiösen und nicht deliranten PatientInnen<br>• bei MA in der Weiterbildung sollte eine Fortsetzung und ggf. Abschluss angestrebt werden.<br>• Assistierende Tätigkeiten<br>• Erstellung eines FEES-Befundes<br>• PatientInnen-/Angehörigengespräch bzgl. Befunde<br>○ Diagnostik und Therapie weiterer Störungsbilder auf Station: Aphasie, Dysarthrie, Apraxie, usw.	○ Trachealkanülen-Management:<br>• (Unter Einhaltung besonderer Schutzmaßnahmen) assistierende Tätigkeiten: Trachealkanülenwechsel, Dekanülierung, Einsetzen/Entfernen von Platzhaltern, Tracheostomapflege<br>• Bei geschlossenen Absaugsystemen besteht keine signifikant höhere Gefahr der Aerosolbildung beim Absaugen über Tubus und Trachealkanüle. Eine Diskonnektion ist zu vermeiden.<br>○ Tätigkeiten, die Aerosolbildung und Körperausscheidungen (Sekret/Speichel) zur Folge haben: Absaugen, Ent-/Aufblocken einer Trachealkanüle, Tubusumlagerung, usw.) nur unter besonderen Schutzmaßnahmen	○ Lagerung/Vertikalisierung (ausgenommen Teillagerungen (bspw. Kopf, Arme))<br>○ Trachealkanülen-Management: Absaugen oral/ endotracheal; Entblocken einer Trachealkanüle, insbesondere bei nicht geschlossenen Systemen<br>○ Trachealkanülenmangement bei inhalativer Sedierung<br>○ Trachealkanülenmanagement und logopädische Therapie insbesondere Dysphagietherapie bei infektiösen oder deliranten PatientInnen<br>○ Videofluroskopie

Atmungstherapeutin

Abgeschlossene Weiterbildungen und Berufserfahrung im Bereich Intensivmedizin sollte als eine Expertise in der Begleitung und Durchführung der nachfolgenden Tätigkeiten verstanden werden.
Empfehlungsgrade: grün = vertretbar, gelb = individuell & eingeschränkt vertretbar, rot = nicht vertretbar
Bei der Bewertung grüner und gelber Bereiche sollte die Expertise und Erfahrung sowie ihr eigenes Sicherheitsempfinden der schwangeren Mitarbeiterin berücksichtigt werden.

**Administrative Aufgaben**		
○ Auswerten und Schreiben von Befunden sowie Überleit-/Entlassbriefen<br>○ Bei bereits etablierten weiteren Tätigkeiten<br>• Abteilungsleitungsfunktionen<br>• Teilnahme an Besprechungen<br>• Mitarbeit in Arbeitsgruppen/Gremien<br>• Arbeit als Medizinproduktebeauftrage<br>• Mitarbeit im klinischen Ethikkomitee<br>○ Mitarbeit bei (Re-)Zertifizierungen (Stroke Unit, Weaning-Zentrum, usw.)<br>○ Erstellen des Weaningprotokolls (in Zusammenarbeit mit dem ärztlichen Dienst)<br>○ Kontrolle und Durchführung der Dokumentation von Beatmungszeiten und Parametern<br>○ Angehörigenkommunikation<br>○ Einbindung in das Verlegungs- und Entlassmanagement (Gespräche mit PatientInnen, nachversorgenden Stellen, Angehörigen, Versorgern, Sozialdienst, usw.)<br>○ Sichtung von Röntgenbildern<br>○ Ggf. Einweisung in Geräte nach MPGD (Medizinprodukte-Durchführungsgesetz) als Medizin Produkte-Beauftragte<br>○ Hausspezifische, patientenferne Tätigkeiten		
**Fort- und Weiterbildung, Forschung und Lehre**		
○ Erarbeitung von Verfahrensanweisung (VA)/ Standard Operating Procedures (SOPs)<br>○ Erstellen und Durchführen von Schulungen von (neuen) Mitarbeitern (Therapie, Pflege, ärztlicher Dienst, Hilfskräfte) zu berufseigenen Themen im Haus<br>○ Supervision und Anleitung sowie Ansprechpartnerin von HospitantInnen, PraktikantInnen		
**Mitwirkung im klinischen Alltag**		
○ Therapieplanung<br>○ Dokumentationsarbeiten: bspw. Erstellen von Berichten<br>○ Angehörigenkommunikation<br>○ Hausspezifische, patientenferne Tätigkeiten<br>○ Blutgasanalyse Auswertung		

Klinische Aufgaben		
○ Maskentraining bei non- invasiv beatmeten PatientInnen<br>○ Inhalationstherapie<br>• mit einem geschlossenen System (Respirator-Schlauchsystem-Patient)<br>• mit Dry Powder Inhaler (DPI) und Metered Dose Aerosol Inhaler (MDI) Devices<br>• Druckluft- und Mesh-Verneblung bei beatmeten PatientInnen ohne Diskonnektion<br>• mit Atemgasbefeuchter (Beatmung, (n-)HFT (High-Flow-Therapy) bei invasiver Beatmung<br>○ Sauerstofftherapie/-titration	○ Non-invasive Beatmung<br>• Bettferne und assistierende Tätigkeiten<br>• Elektive Einstellung und Anpassung<br>○ Weaning<br>• Spontanatemversuche (mit Diskonnektion vom Respirator) (mit höherer Exposition für Aerosolbildung und Körperausscheidungen) → nur unter Einhaltung besonderer Schutzmaßnahmen<br>○ Blutgasanalyse kapillär und Abnahme über einen liegenden Zugang („Arterie)<br>○ Atemtherapeutische Tätigkeiten<br>• Bei Atemübungen (mit/ohne Hilfsmittel) ohne Aerosolbildung & entsprechenden Schutzmaßnahmen<br>• Atemtrainer, mit Ziel Sekret zu fördern, sollten nicht genutzt werden<br>○ Trachealkanülenmanagement<br>• ohne Aerosolbildung & entsprechenden Schutzmaßnahmen<br>• Trachealkanülenwechsel, Dekanülierung, Einsetzen/Entfernen von Platzhaltern, Tracheostomapflege<br>• Ent-/ Aufblocken einer Trachealkanüle; Tubusumlagerung<br>○ Atemwegsmanagement<br>• Vor- und Nachbereitung bei Tracheotomien sowie Bronchoskopien<br>○ Absaugen mit geschlossenem Absaugsystemen über Tubus und Trachealkanülen	○ Lagerung, Vertikalisierung<br>○ Körperliche Anstrengung<br>○ Tätigkeiten bei infektiösen PatientInnen<br>○ Non-invasive Beatmung<br>• Bei Akutfällen (bspw. exazerbierte chronisch obstruktive Lungenerkrankung (COPD), Pneumonie, kardiales Lungenödem)<br>• Umstellung von invasiver auf non-invasive Beatmung<br>○ Atemwegsmanagement<br>• Intubation, Extubation, Tracheotomie (bedside, dilatativ), Bronchoskopien<br>○ Titration von Heimbeatmungen (invasiv/non-invasiv)<br>○ Titration von Cough Assist<br>○ Sekretmanagement (bspw. Sekretfördernde, manuelle Maßnahmen und Cough Assist)<br>○ Druckluft- und Mesh-Verneblung<br>○ Inhalationstherapie mit Atemgasbefeuchter (Beatmung (n-)HFT (High-Flow-Therapy)) bei non-invasiver Beatmung und Spontanatmung<br>○ Arbeiten bei inhalativer Sedierung<br>○ Absaugen oral und endotracheal bei offenen Systemen<br>○ Trachealkanülenmanagement<br>• mit Aerosolbildung
